# Age-associated reduction of cellular spreading/mechanical force up-regulates matrix metalloproteinase-1 expression and collagen fibril fragmentation via c-Jun/AP-1 in human dermal fibroblasts

**DOI:** 10.1111/acel.12265

**Published:** 2014-09-08

**Authors:** Zhaoping Qin, John J Voorhees, Gary J Fisher, Taihao Quan

**Affiliations:** Department of Dermatology, University of Michigan Medical SchoolAnn Arbor, Michigan, USA

**Keywords:** aging, c-Jun/AP-1, extracellular matrix microenvironment, fibroblast, mechanical force, MMP-1

## Abstract

The dermal compartment of human skin is largely composed of dense collagen-rich fibrils, which provide structural and mechanical support. Skin dermal fibroblasts, the major collagen-producing cells, are interact with collagen fibrils to maintain cell spreading and mechanical force for function. A characteristic feature of aged human skin is fragmentation of collagen fibrils, which is initiated by matrix metalloproteinase 1 (MMP-1). Fragmentation impairs fibroblast attachment and thereby reduces spreading. Here, we investigated the relationship among fibroblast spreading, mechanical force, MMP-1 expression, and collagen fibril fragmentation. Reduced fibroblast spreading due to cytoskeletal disruption was associated with reduced cellular mechanical force, as determined by atomic force microscopy. These reductions substantially induced MMP-1 expression, which led to collagen fibril fragmentation and disorganization in three-dimensional collagen lattices. Constraining fibroblast size by culturing on slides coated with collagen micropatterns also significantly induced MMP-1 expression. Reduced spreading/mechanical force induced transcription factor c-Jun and its binding to a canonical AP-1 binding site in the MMP-1 proximal promoter. Blocking c-Jun function with dominant negative mutant c-Jun significantly reduced induction of MMP-1 expression in response to reduced spreading/mechanical force. Furthermore, restoration of fibroblast spreading/mechanical force led to decline of c-Jun and MMP-1 levels and eliminated collagen fibril fragmentation and disorganization. These data reveal a novel mechanism by which alteration of fibroblast shape/mechanical force regulates c-Jun/AP-1-dependent expression of MMP-1 and consequent collagen fibril fragmentation. This mechanism provides a foundation for understanding the cellular and molecular basis of age-related collagen fragmentation in human skin.

## Introduction

Human skin is composed of a dense collagen-rich connective tissue that provides structural and mechanical support (Uitto, 1986). This collagen-rich connective tissue is produced, organized, and maintained by dermal fibroblasts. During aging, dermal connective tissue undergoes progressive alterations in part associated with age-related collagen fibril fragmentation and disorganization (Fisher *et al*., 2008, 2009). This age-dependent alteration of collagen fibrils is largely driven by elevated expression of matrix metalloproteinase one (MMP-1) (Fisher *et al*., 2009), which initiates cleavage of collagen fibrils in skin. Alterations of dermal connective tissue collagen impair skin structural integrity and are associated with age-related disorders such as delayed wound healing (Uitto, 1986) and cancer (Bissell *et al*., 2005; Bissell & Hines, 2011; Lu *et al*., 2012).

In human dermis, fibroblasts are embedded in a collagen-rich microenvironment and physically interact with collagen fibrils to maintain normal cell shape and mechanical force. Accumulating evidence indicates that cell shape and mechanical force regulate essential cell functions (Hynes, 2009). Cell shape and mechanical force largely rely on interactions of cells with surrounding extracellular matrix (ECM). The ECM must provide both binding sites for cells and mechanical resistance to cellular traction forces (Ingber, 2006; Geiger *et al*., 2009). In young healthy skin, dermal fibroblasts attach to intact collagen fibrils and exert traction forces to achieve normal cell shape and mechanical tension. However, in aged dermis, collagen fibrils are fragmented, which reduces both fibroblast binding and mechanical stability. These alterations impair fibroblast spreading, mechanical forces, and functions (Varani *et al*., 2004; Fisher *et al*., 2008; Quan *et al*., 2013b). While cell shape and mechanical forces are known to regulate many cellular functions, the molecular basis of their impact on dermal fibroblast function and skin connective tissue aging is not well understood.

We previously reported that dermal fibroblasts in aged human skin expresses elevated MMP-1, which leads to collagen fibril fragmentation and disorganization, the hallmark of skin connective tissue aging (Fisher *et al*., 2008; Quan *et al*., 2013a). We also reported that a prominent characteristic of dermal fibroblasts in aged skin is reduced spreading and contact with collagen fibrils, resulting in collapsed morphology; cells lose their typical elongated spindle-like morphology and become shorter with a rounded and collapsed morphology (Varani *et al*., 2004; Fisher *et al*., 2008, 2009; Quan *et al*., 2013a,b2013b). These data prompted us to explore the connection between reduced spreading/mechanical force and elevated MMP-1 in dermal fibroblasts.

We report that reduced fibroblast spreading and mechanical force activates transcription factor c-Jun/AP-1 leading to up-regulation of MMP-1 expression. Our data provide insights into mechanisms that likely operate to promote collagen fibril fragmentation, which is a defining feature of the pathophysiology of human skin aging.

## Results

### Reduced fibroblast spreading decreases mechanical force and induces MMP-1

**Figure 1:**
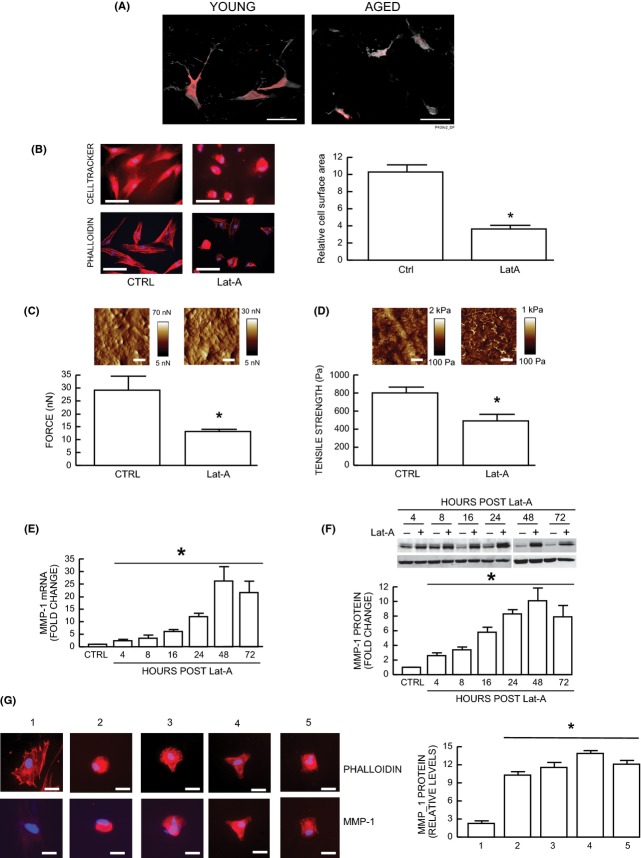
Reduced spreading/mechanical force induces MMP-1 expression in human skin dermal fibroblasts. (A) Representative images of dermal fibroblasts in aged (75 years, right panel) and young (24 years, left panel) human skin. Skin was sectioned, cells were stained with HCS Cell Mask Deep Red fluorescent dye (gray), and fibroblasts were identified by immunostaining with collagen chaperone heat-shock protein 47 (red). Note spread fibroblasts in young skin vs. contracted fibroblasts in aged human skin. Images were obtained by multiphoton laser scanning fluorescence microscopy. *N* = 10 for each group. Bars = 25 μm. (B–D) Dermal fibroblasts were treated with Lat-A (30 nm) for 24 h. (B) Dermal fibroblasts were stained with CellTracker® fluorescent dye or phalloidin and were imaged by fluorescence microscopy. Red fluorescence delineates cell cytoplasm; blue fluorescence delineates nuclei. The relative cell surface areas were quantified by ImageJ. Mean ± SEM, *N* = 6, *P < 0.05. Bars = 100 μm. (C) Cell traction force (nN) and (D) tensile strength (Pa) were determined by AFM PeakForce Quantitative NanoMechanics mode and analyzed by Nanoscope Analysis software as described in *Methods*. Mean ± SEM. Bars = 100 nm, *N* = 6, *P < 0.05. Insets show representative images of traction force and tensile strength. Vertical bars indicate traction forces (nN) and tensile strength (Pa) scales. (E) MMP-1 mRNA levels were quantified by real-time RT–PCR at indicated times after Lat-A (30 nm) treatment. MMP-1 mRNA levels were normalized by the housekeeping gene (36B4, internal control). Mean ± SEM. *N* = 3–12, *P < 0.05. (F) MMP-1 protein levels were determined by Western blot analysis at indicated times after Lat-A (30 nm) treatment. MMP-1 protein levels were normalized by and β-actin (loading control). Inset shows representative Western blot. Mean ± SEM, *N* = 4–5, *P < 0.05. (G) Cells were cultured on type I collagen-coated microplates with nonpatterned/full adhesive (#1) and micropatterns of different shapes (#2–#5) and size (approximately one-third the size of #1, nonpatterned/full adhesive). #1, CTRL (nonpatterned, fully spread); #2, Disk shape; #3, Crossbow shape; #4, Y shape; #5, I shape. MMP-1 protein levels were determined by immunohistology and the relative levels were quantified using ImageJ software. Mean ± SEM, *N* = 4, *P < 0.05. Bars = 100 μm.

Multiphoton fluorescence microscopy illustrates the contracted morphology of dermal fibroblasts in aged human skin (Fig.1A right panel), compared with the stretched appearance of fibroblasts in young skin (Fig.1A left panel). Based on these human skin *in vivo* observations, we investigated whether reduced spreading and mechanical force contribute to impaired dermal fibroblast function in aged human skin. We modulated dermal fibroblast shape and mechanical force by disrupting the actin cytoskeleton with latrunculin-A (Lat-A), which rapidly blocks actin polymerization (Gieni & Hendzel, 2008). As expected, disruption of the actin cytoskeleton reduced fibroblast spreading. The cells acquired a rounded shape lacking extensions (Fig.1B, upper right panel). Staining of the actin cytoskeleton with phalloidin further indicated loss of actin cytoskeletal fibers and reduced cell area (Fig.1B, lower right panel). Fibroblast area was reduced approximately 70% (Fig.1B).

We next employed quantitative nanomechanical mapping method of atomic force microscopy (AFM)(Thomas *et al*., 2013) to determine changes in mechanical properties associated with reduced fibroblast spreading. AFM indicated that the key cellular mechanical properties of traction force (Fig.1C) and tensile strength (Fig.1D) were significantly reduced by 49% and 40%, respectively. Importantly, reduced fibroblast spreading/mechanical force was associated with time-dependent increase of MMP-1 mRNA (Fig.1E) and protein (Fig.1F) levels. MMP-1 expression was elevated within 4 h, continued to rise during 48 h and remained elevated for at least 72 h. Elevated MMP-1 expression is a prominent feature of dermal fibroblasts in aged human skin (Fisher *et al*., 2008, 2009; Quan *et al*., 2013a).

The above data indicate that reduced fibroblast spreading, due to disassembly of the actin cytoskeleton, leads to elevated MMP-1 expression. We next investigated the relationship between fibroblast spreading, actin cytoskeleton assembly, and MMP-1 protein expression. We used glass substrates whose surface was coated with patterned arrays of discrete spots of type I collagen. These spots served as attachment sites for fibroblasts, which otherwise could not attach to the glass surface. The spots were spaced to create four different geometric shapes of specific size, which was approximately one-third the size of fully spread fibroblasts. Thus, attachment to the type I collagen spots determined fibroblast size and shape. Reducing fibroblast size, without impairing actin cytoskeleton assembly, resulted in significant induction of MMP-1 protein expression (Fig.1G). This induction was observed regardless of fibroblast shape, which included circle, diamond, triangle, and square.

### Fragmentation of collagen fibrils by fibroblasts in three-dimensional collagen lattices

**Figure 2:**
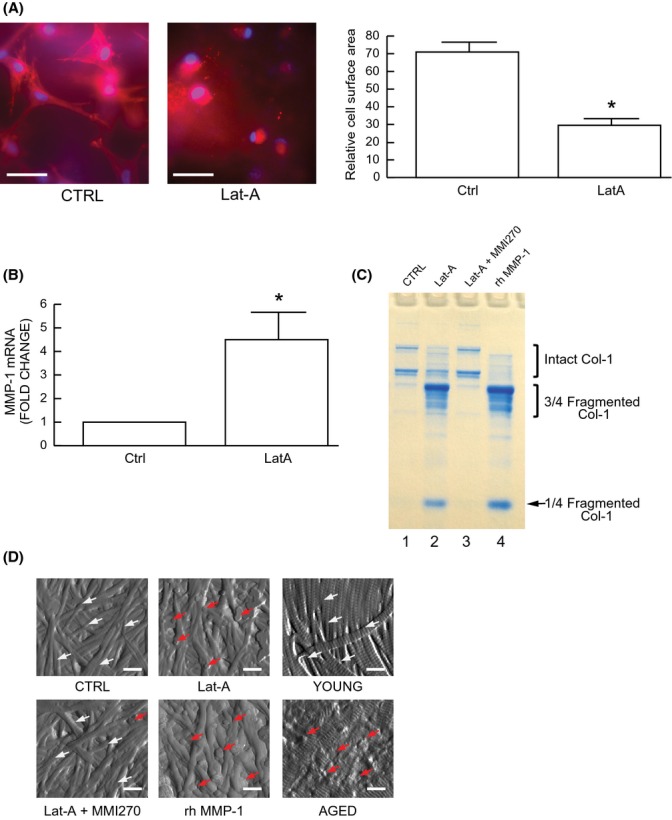
Induction of MMP-1 in human dermal fibroblasts by reduced spreading/mechanical force stimulates collagen fragmentation. (A–D) Dermal fibroblasts were treated with Lat-A (30 nm) for 24 h. (A) Dermal fibroblasts were cultured in 3D type I collagen gels as described in Methods. Collagen gels were stained with CellTracker® fluorescent dye. Red fluorescence delineates cell cytoplasm; blue fluorescence delineates nuclei. Relative cell surface areas were quantified using ImageJ software. Mean ± SEM, *N* = 3, *P < 0.05. Bars = 100 μm. (B) MMP-1 mRNA levels were quantified by real-time RT–PCR. MMP-1 mRNA levels were normalized by the housekeeping gene (36B4, internal control). Mean ± SEM. *N* = 6, *P < 0.05. (C) Conditioned media were collected, concentrated, and resolved by 10% SDS–PAGE. Intact and fragmented collagens were visualized by staining with SimplyBlue. MMPs inhibitor (MMI270) and activated recombinant human MMP-1 (rhMMP-1) were used as negative and positive controls, respectively. *N* = 3. (D) Nanoscale collagen fibrils were visualized by AFM using ScanAsyst mode, as described in *Methods*. Sun-protected buttocks human skin samples were obtained from young (21–30 years) and aged (80 + years) individuals. White arrows indicate intact and organized collagen fibrils, and red arrows indicate damaged and disorganized collagen fibrils. Bars = 200 nm. *N* = 6.

In skin, fibroblasts are embedded within the dermal collagen fibril-rich three-dimensional extracellular matrix. Therefore, we next cultured dermal fibroblasts in three-dimensional collagen lattices to examine the functional consequences of elevated MMP-1 expression resulting from reduced spreading/mechanical force. As observed in monolayer culture (Fig.1B, right panel), disruption of the actin cytoskeleton also caused reduced spreading (Fig.2A). In collagen lattice cultures, fibroblast size was reduced by approximately 60% (Fig.2A). This reduced spreading significantly increased MMP1 expression (Fig.2B) and generated one quarter and three-quarter length collagen fragments (Fig.2C lane 2), which are characteristic of MMP-1 activity (Gross *et al*., 1974; Fields, 1991), as shown by treatment of lattices with recombinant human MMP-1 (Fig.2C lane 4). Fibroblast-mediated collagen fibril fragmentation was completely blocked by a MMP inhibitor (MMI270) (Fig.2C lane 3). AFM images revealed that collagen fibrils in lattices containing untreated fibroblasts were intact and well-organized, displaying characteristic d-bands (Fig.2D, upper left panel). In contrast, collagen fibrils in lattices containing fibroblasts with reduced spreading, due to cytoskeletal disruption, were fragmented and disorganized (Fig.2D, upper middle panel), similar to collagen fibrils in lattices treated with recombinant human MMP-1 (Fig.2D, lower middle panel). Fibroblast-mediated collagen fibril disruption was blocked by a MMP inhibitor (MMI270) (Fig.2D, lower left panel). For comparison, AFM images of collagen fibrils in young and aged human skin are shown in the right panels of Fig.2D. It can be seen that collagen fibrils in lattices containing untreated fibroblasts (Fig.2D, upper left panel) resemble those in young skin (Fig.2D, upper right panel). In contrast, collagen fibrils in lattices containing fibroblasts with reduced spreading (Fig.2D, upper middle panel) resemble those in aged human skin (Fig.2D, lower right panel). These data demonstrate that reduced fibroblast spreading/cytoskeletal tension increases MMP-1 expression and generates collagen fragmentation and disorganization, as observed in aged human skin *in vivo*.

### Induction of c-Jun/AP-1 mediates up-regulation of MMP-1 in response to reduced fibroblast spreading/mechanical force

**Figure 3:**
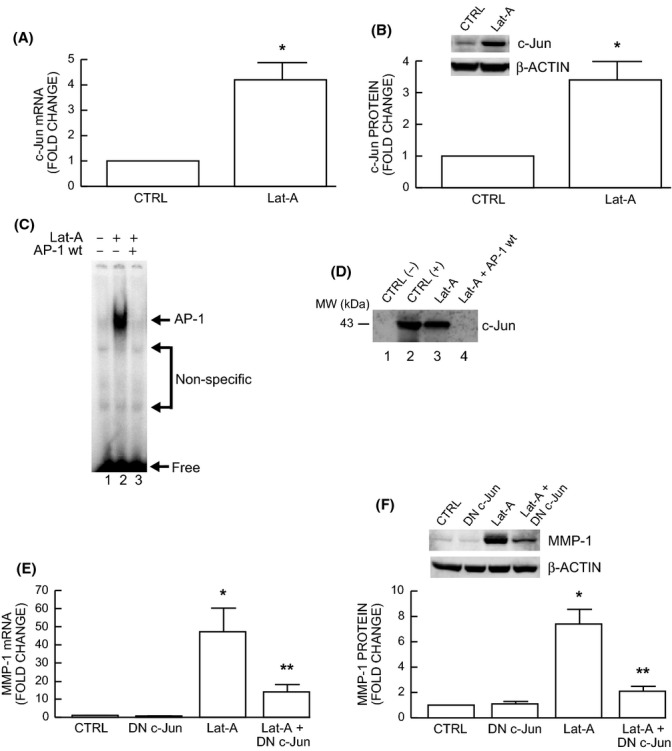
Induction of c-Jun/AP-1 in human dermal fibroblasts by reduced spreading/mechanical force mediates up-regulation of MMP-1. (A-F) Fibroblasts were treated with Lat-A (30 nm) for 24 h. (A) c-Jun mRNA levels were quantified by real-time RT–PCR. c-Jun mRNA levels were normalized by the housekeeping gene (36B4, internal control) Mean ± SEM, *N* = 6, *P < 0.05. (B) c-Jun protein levels were determined by Western blot analysis, and relative levels were normalized by and β-actin (loading control). Inset shows representative Western blots. Mean ± SEM, *N* = 6, *P < 0.05. (C) Protein binding to the AP-1 binding site of the MMP-1 promoter was determined by electrophoretic mobility shift assays (EMSA). *N* = 3. (D) Identification of c-Jun proteins in MMP-1 promoter was determined by EMSA coupled with Western blot analysis. *N* = 3. (E) Functional blocking of c-Jun inhibits induction of MMP-1 mRNA by reduced spreading/mechanical force. Cells were transfected with dominant negative c-Jun prior to treatment with Lat-A. MMP-1 mRNA levels were quantified by real-time RT–PCR. MMP-1 mRNA levels were normalized by the housekeeping gene (36B4, internal control). Mean ± SEM, *N* = 3, *P < 0.05 vs. CTRL. **P < 0.05 vs. Lat-A. (F) Functional blocking of c-Jun inhibits MMP-1 protein by reduced spreading/mechanical force. Fibroblasts were transfected with dominant negative c-Jun (DN c-Jun) prior to treatment with Lat-A. MMP-1 protein levels were determined by Western blot analysis. MMP-1 protein levels were normalized by β-actin (loading control). Inset shows representative Western blots. Mean ± SEM, *N* = 3, *P < 0.05 vs. CTRL. **P < 0.05 vs. Lat-A.

We next investigated the mechanisms by which reduced fibroblast spreading/mechanical force up-regulates MMP-1 expression. We focused on transcription factors c-Jun and c-Fos, which are typically comprise the AP-1 transcription factor complex, and has been shown to principal regulator of MMP-1 transcription, under a variety of conditions (Gutman & Wasylyk, 1990; Birkedal-Hansen *et al*., 1993; Mauviel, 1993). As shown in Fig.3, reduced spreading/mechanical force significantly increased c-Jun mRNA (Fig.3A) and protein (Fig.3B). c-Fos mRNA was similarly induced (4.6 fold ± 1.5, *N* = 4, data not shown). Furthermore, electrophoretic mobility shift assays (EMSA) indicated that reduced spreading/mechanical force markedly increased protein binding to the AP-1 binding site (5′-TAAAGCATGAGTCAGACAC-3′) of the MMP-1 promoter (Fig.3C, lane 2). This retarded complex formed with the MMP-1 probe was completely abolished by excess unlabeled wild-type AP-1 probe (Fig.3C, lane 3). To confirm that the retarded complex contained c-Jun protein, we performed EMSA coupled with Western blot (Quan *et al*., 2005). The regions of the gel containing retarded complexes that were induced in response to reduced spreading/mechanical force were excised, and protein was extracted and subjected to SDS-PAGE immunoblotting with antibody to c-Jun. c-Jun protein was readily detected from the retarded complexes formed with the MMP-1 probe (Fig.3D, lane 3) but not from the control probe (Fig.3D, lane 1) and wild-type AP-1 probe competition (Fig.3D, lane 4).

We next examined whether interfering with c-Jun function could block induction of MMP-1 in response to reduced spreading/mechanical force. Dermal fibroblasts were transfected with a well-characterized dominant negative c-Jun (Li *et al*., 2000; Quan *et al*., 2010) prior to treatment with Lat-A. MMP-1 mRNA (Fig.3E) and protein (Fig.3F) were significantly reduced by blocking c-Jun function. Taken together, the above data demonstrate that reduced spreading/mechanical force increases c-Jun/AP-1, which mediated induction of MMP-1 in human dermal fibroblasts.

### Restoration of lost cell shape/mechanical tension reversed elevated c-Jun, MMP-1, and collagen fragmentation/disorganization

**Figure 4:**
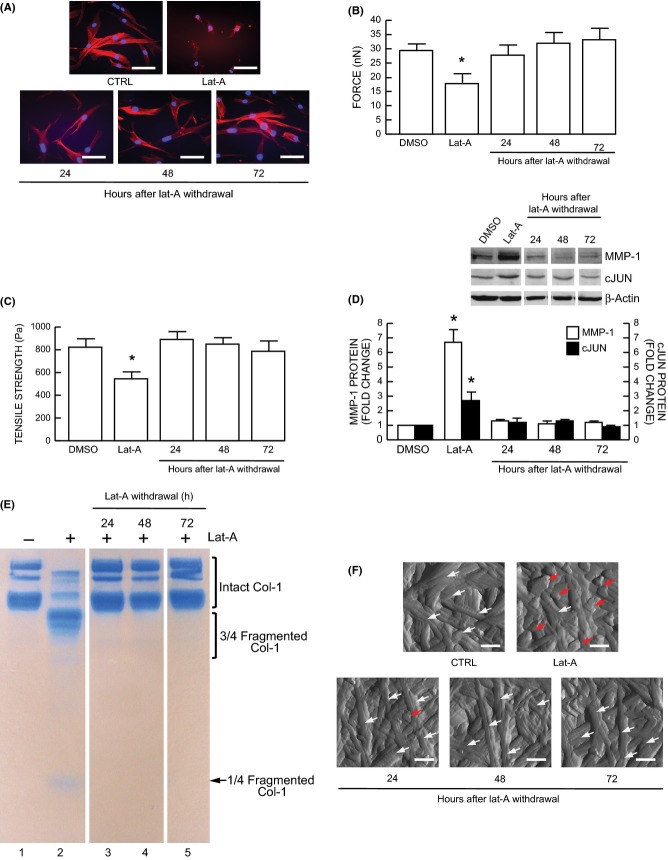
Restoration of dermal fibroblast spreading/mechanical force reverses elevated MMP-1, c-Jun, and collagen fragmentation/disorganization. Lat-A was withdrawn 24 h after Lat-A (30 nm) treatment by replacing with fresh culture medium, and the cells were further incubated for the indicated times. (A) Dermal fibroblasts were stained with phalloidin and imaged by fluorescence microscopy. Red fluorescence delineates cell cytoplasm; blue fluorescence delineates nuclei. Bars = 100 μm. *N* = 6. (B) Cell traction forces (nN) and (C) tensile strength (Pa) were determined using AFM PeakForce Quantitative NanoMechanics mode and analyzed using Nanoscope Analysis software, as described in *Method*. Means ± SEM. *N* = 6, *P < 0.05. (D) c-Jun and MMP-1 protein levels were determined by Western blot analysis. c-Jun and MMP-1 protein levels were normalized by β-actin (loading control). Inset shows representative Western blots. Mean ± SEM, *N* = 3–6, *P < 0.05. (E) Conditioned media were collected, concentrated, and resolved by 10% SDS–PAGE. Intact and fragmented collagens were visualized by staining with SimplyBlue. *N* = 3. (F) Nanoscale collagen fibrils were visualized using AFM ScanAsyst mode as described in *Methods*. White arrows indicate intact and organized collagen fibrils, and red arrows indicate damaged and disorganized collagen fibrils. Bars = 200 nm. *N* = 3.

Finally, we assessed whether elevated c-Jun/MMP-1 and collagen fragmentation/disorganization are reversible by restoration of fibroblast spreading/mechanical force. For these studies, Lat-A-containing media was withdrawn 16–24 h after its addition, the cells were extensively washed, and fresh culture media was added. With removal of Lat-A, fibroblasts converted from a small, rounded morphology (Fig.4A upper right panel) to typical elongated morphology (Fig.4A, lower panels) and recovered cellular mechanical properties of traction force (Fig.4B) and tensile strength (Fig.4C). Consistent with recovery of cellular morphology/mechanical force, elevated c-Jun and MMP-1 were reduced to basal level (Fig.4D). Furthermore, MMP-1-generated collagen fragmentation (Fig.4E, lane 2) was substantially reduced, and collagen fibril structure and organization (Fig.4F, upper right panel) in three-dimensional collagen lattices were normalized following restoration of fibroblast spreading/mechanical force.

## Discussion

Collagen fibril fragmentation is considered an important process in human skin connective tissue aging. Fragmentation is in part initiated by MMP-1, which becomes elevated during the aging process in human skin (Fisher *et al*., 2008, 2009). We demonstrate here that reduced fibroblast spreading/mechanical tension up-regulates MMP-1 expression as observed in aged human skin dermal fibroblasts *in vivo* (Fisher *et al*., 2008, 2009; Quan *et al*., 2013a). Reduced cell spreading is a prominent feature of dermal fibroblasts in aged human skin (Varani *et al*., 2004; Fisher *et al*., 2008, 2009). These data suggest that elevated MMP-1 expression in aged human skin arises, at least in part, from reduced spreading/mechanical force of dermal fibroblasts. In contrast to our findings, Kook *et al*. recently reported that increased tensile force, by application of vacuum stretch to periodontal ligament fibroblasts on flexible plates, resulted in transient activation of JNK-AP-1 and induction of MMP-1 (Kook *et al*., 2011). Although the mechanisms responsible for differences between our results and those of Kook *et al*. are not clear, it is conceivable that they reflect different experiment conditions and/or tissue specificity.

In skin dermis, fibroblast spreading, which is mediated by cytoskeletal and intracellular structural machinery, largely depends on cellular interactions with surrounding ECM microenvironment. In young human skin dermis, binding of fibroblasts to intact collagen fibrils allows generation of traction forces that are necessary for spreading, mechanical stability, normal function. However, in aged human skin dermis, collagen fibril binding sites are lost and mechanical resistance to traction forces is reduced due to fragmentation. In this state, the ECM microenvironment is unable to provide sufficient mechanical stability to maintain normal cell spreading/mechanical force. Therefore, age-related fragmentation of the collagen fibril microenvironment deleteriously alters fibroblast morphology and function.

We recently demonstrated that increasing structural and mechanical properties of the dermal microenvironment can activate fibroblasts to a more ‘youthful’ state in aged human skin *in vivo* (Wang *et al*., 2007; Quan *et al*., 2012, 2013b). Injection of dermal filler (cross-linked hyaluronic acid) into the skin of individuals over 70 years of age stimulates fibroblast collagen production and proliferation, expands vasculature, and increases epidermal thickness. This stimulation is associated with localized increase of mechanical forces and enhancement of the structural integrity as indicated by fibroblast spreading and new collagen deposition. Thus, fibroblasts in aged human skin retain their capacity for functional activation, which can be restored by enhancing structural and mechanical support of the ECM. These data also indicate that proliferation and function of other cell types, including endothelial cells and keratinocytes, can be enhanced in aged skin by increased structural support of dermal ECM microenvironment. These findings support the concept that cell spreading/mechanical force, along with tissue microenvironment, are critical for dermal fibroblast function. Furthermore, these findings extend current knowledge of mechanisms of skin aging beyond intrinsic cellular processes to include the dermal ECM microenvironment.

Further investigation demonstrated that reduced spreading/mechanical tension induced c-Jun and AP-1 activity. This activation resulted in increased MMP-1 expression, which is known to be transcriptionally regulated by AP-1 (Gutman & Wasylyk, 1990; Birkedal-Hansen *et al*., 1993; Mauviel, 1993). Transcription factor AP-1, typically composed of c-Jun and c-Fos, is one of the first mammalian transcription factors to be identified (Angel & Karin, 1991; Shaulian & Karin, 2002). To our knowledge, this is the first demonstration that AP-1 activity is induced in response to reduced cellular spreading/mechanical tension. In human skin, AP-1 activity is limited by the low level of c-Jun, whereas c-Fos is constitutively expressed (Fisher *et al*., 2000). We and others previously reported that stress-activated MAP Kinase pathways and c-Jun mRNA and protein are increased in aged, compared with young human skin *in vivo* (Chung *et al*., 2000; Shin *et al*., 2005; Fisher *et al*., 2009). These data suggest that reduced cellular spreading/mechanical force induces c-Jun, which in turn elevates MMP-1 expression in aged human skin. Additionally, we and others have reported that AP-1 negatively regulates type I procollagen expression (Fisher *et al*., 2000; Verrecchia *et al*., 2000). Therefore, elevated AP-1 appears plays an important role in human skin connective tissue aging.

Cell shape/mechanical force impacts multiple cellular processes including signal transduction, gene expression, and metabolism (Wang *et al*., 1993; Silver *et al*., 2003; Alenghat *et al*., 2004; Ingber, 2006). Currently, mechanisms by which altered cell spreading/mechanical force induces c-Jun/AP-1 are not well understood. AP-1 activity is regulated by a wide range of stimuli including reactive oxygen species (ROS) (Shaulian & Karin, 2002). We previously reported that fibroblasts that have reduced spreading/mechanical force due to fragmentation of surrounding collagen fibrils display increase levels of ROS (Fisher *et al*., 2009). ROS is considered to be a major driving force for the aging process (Stadtman, 1992; Golden *et al*., 2002). Indeed, we observed that protein oxidation, a marker of oxidative stress, is increased in aged human dermis *in vivo* (Fisher *et al*., 2009). These data suggest that elevated ROS may be mediate induction of c-Jun and AP-1 activity in response to reduced fibroblast spreading/mechanical force. Further studies are needed to understand the mechanisms that couple fibroblast spreading/mechanical force to oxidative stress and the role of these mechanisms in skin connective tissue aging.

We have previously reported that the primary dermal fibroblasts from aged (>80 years) or young (>30 years) individuals, which are cultured in monolayer, in tissue culture plastic dishes, are indistinguishable from each other with respect to morphology and a variety of molecular endpoints, including levels of MMP-1, type I collagen, and key regulatory pathways including c-Jun/AP-1, ROS and TGF-β signaling (Fisher *et al*., 2009). In contrast, human dermal fibroblasts, obtained from individuals of any age (21–86 years of age), cultured in three-dimensional collagen lattices that have been partially fragmented by treatment with exogenous MMP-1, have a reduced spreading and contracted appearance, consistent with reduced mechanical tension. These ‘collapsed’ fibroblasts have elevated levels of MMP-1 and reduced collagen production, compared with ‘stretched’ fibroblasts cultured in intact collagen lattices. These observations suggest that the collagenous ECM microenvironment of the dermis is largely responsible for the morphological and functional alterations that fibroblasts undergo in during aging in human skin *in vivo*.

Elevated MMP-1 in aged skin breaks down collagen fibrils, thereby weakening the structural integrity and impairing attachment of fibroblasts to the dermal extracellular matrix. These conditions cause reduced fibroblast spreading and mechanical force. This reduced cell spreading/mechanical tension further stimulates MMP-1 through activation of c-Jun/AP-1. The positive feedback (or vicious cycle) relationship between elevated MMP-1 and reduced cell spreading/mechanical tension is consistent with the biology of aging, which is epitomized by continual reduction of homeostatic control.

In summary, this study demonstrates that reduced fibroblast spreading/mechanical force substantially up-regulates MMP-1 expression, which causes collagen fibril fragmentation, a prominent feature of aged human skin. Up-regulation of MMP-1 is mediated by induction c-Jun and AP-1 activity. Therefore, targeting AP-1 may improve the structure and function of aged human skin, thereby mitigating age-related skin diseases.

## Experimental procedures

### Cell culture

Primary adult human dermal fibroblasts were prepared from 4-mm full-thickness punch biopsies of healthy volunteers (mean age 31 ± 4 years) as previously described (Fisher *et al*., 1991). Briefly, human skin biopsies were obtained from sun-protected buttock skin, and dermal fibroblasts were isolated by digestion skin with bacterial collagenase (Worthington Biochemical Corporation, Lakewood, NJ, USA). Early passage (<9 passages) primary adult human dermal fibroblasts were cultured in Dulbecco's modified Eagle's media (DMEM, Invitrogen Life Technology, Carlsbad, CA, USA) with 10% fetal calf sera (FBS, Invitrogen Life Technology) at 37 °C, 5% CO_2_. Approximate population doubling time is 2 days. In some case, cells were cultured on type I collagen-coated microplates (CYTOOchips™Mini, CYTOO Inc, Cambridge, MA, USA) with adhesive micropatterns of different sizes and shapes. A microplate is a 20 mm × 20 mm, 175-μm-thick gridded coverslip, which contains 12 different micropatttens: three different sizes (small, medium, and large) and four different shapes (disk, crossbow, I and Y shapes). These micropatterns are coated with type I collagen to form adhesive substrates of defined size and shape surrounded by a cytophobic surface. Each specific size/shape is composed of 1728 identical micropatterns. 3D collagen gels were prepared based on a previous publication with minor modification (Qin *et al*., 2014). Briefly, neutralized rat tail type I collagen (2 mg/mL, BD, Biosciences, Palo Alto, CA, USA) was suspended in medium cocktail (DMEM, NaHCO3 [44 mm], L-glutamine [4 mm], folic acid [9 mm], and neutralized with 1N NaOH to pH 7.2). Cells (0.5 × 10^6^) were suspended in 2 mL collagen and medium cocktail solution and plated in a 35-mm bacterial culture dish. The collagen gels were placed in incubator at 37 °C for 30 min to allow collagen polymerization. The collagen gels were then incubated with 2 mL media (DMEM, 10% FBS) at 37 °C, 5% CO_2_. For latrunculin-A (Lat-A) treatment, cells were treated with Lat-A at a concentration of 30 nm for 24 h or the indicated times. To activate secreted MMP-1, collagen lattices were washed extensively with PBS (at least three times for 30 min) and then treated with Trypsin-EDTA (100 ng/mL, Invitrogen Life Technology) in serum-free media for 24 h. Conditioned media were collected, concentrated, and analyzed by 10% SDS–PAGE. Collagen bands were visualized by staining with SimplyBlue (Invitrogen Life Technology).

### RNA isolation and quantitative real-time RT–PCR

RNA isolation and quantitative real-time RT–PCR were performed as described previously (Quan *et al*., 2011). Briefly, total RNA was extracted by Trizol reagent, and 100 ng total RNA was reverse transcribed using a Taqman Reverse Transcription kit (Applied Biosystems, Foster City, CA, USA). Real-time RT–PCR was performed by SYBR green real-time PCR using a 7300 Sequence Detector (Applied Biosystems). Human MMP-1, c-Jun, and 36B4 primers were described previously (Qin *et al*., 2014). Target gene mRNA was normalized to the housekeeping gene 36B4 (a ribosomal protein used as an internal control for quantitation) as an internal control.

### Transfection and Western blotting

For transfection, primary adult human dermal fibroblasts were transiently transfected with dominant negative c-Jun (Li *et al*., 2000) by electroporation (Amaxa Biosystems, Gaithersburg, MD). For Western blot analysis, cells were lyzed in whole cell extraction buffer (25 mm HEPES [pH 7.7]; 0.3 m NaCl; 1.5 mm MgCl_2_; 0.2 mm EDTA; 0.1% Triton X-100; 0.5 mm DTT' 20 mm β-glycerolphosphate; 0.1 mm Na_3_VO_4_; 2 μg/mL leupeptin; and 100 μg/mL PMSF) followed by centrifugation. Protein concentration was determined by the Bio-Rad protein assay (Bio-Rad laboratories, Hercules, CA, USA) using bovine serum albumin as a standard. Whole cell extracts (30–50 μg) were resolved by 10% SDS-PAGE, transferred to PVDF membrane, and blocked with PBST (0.1% Tween 20 in PBS) containing 5% milk for 1 h at room temperature. The primary antibodies against MMP-1 and c-Jun (Santa Cruz Biotechnology, Santa Cruz, CA, USA) were incubated with PVDF membrane for 1 h at room temperature. Blots were washed three times with PBST solution and incubated with appropriate secondary antibody for 1 h at room temperature. After washing three times with PBST, the blots were developed with ECF (Vistra ECF Western Blotting System, Amersham Pharmacia Biotech, Piscataway, NJ, USA) following the manufacturer's protocol (Molecular Dynamics, Sunnyvale, CA, USA). The blots were scanned by a STORM PhosphorImager, and intensities of each band were normalized to β-actin (Sigma, St. Louis, MO, USA) as an internal control.

### CellTracker, Phalloidin staining, Immunohistology, and second harmonic generation microscopy

Cell morphology was assessed by incubation of cultures with CellTracker® fluorescent dye (Molecular Probes, Eugene, OR, USA) for 1 h. The cells were washed with PBS and were fixed in 2% paraformaldehyde for 30 min. For Phalloidin staining, cells were washed with PBS and were fixed in 2% paraformaldehyde for 30 min followed by Phalloidin stain (Sigma) for 1 h. For immunohistology, cryosections (7 μm thickness) were fixed in 2% paraformaldehyde for 2 h at room temperature and were incubated with 0.5% Nonidet P-40, then blocked with 2% bovine serum albumin (BSA). The slides were washed with PBS five times and incubated with MMP-1 and HSP47 primary antibodies (Santa Cruz Biotechnology) for 1 h at room temperature. Cells were washed and then incubated with secondary antibody for 30 min at room temperature. Images were obtained using Zeiss fluorescence microscopy. Second harmonic generation microscopy was performed using a Leica SP8 Confocal Microscope with 2-Photon, at University of Michigan Microscopy and Image Analysis Laboratory.

### Electrophoretic mobility shift assay (EMSA)

EMSA was performed as described previously (Quan *et al*., 2001). Briefly, nuclear extracts were prepared using Nuclear and Cytoplasmic Extraction reagents (Pierce, Rockford. IL, USA). Double-stranded oligodeoxynucleotides containing the AP-1 binding site in the MMP-1 promoter (5′-ATAAAGCA**TGAGTCA**GACAGCTC-3′) were used as probe. The AP-1 binding site is in bold font. All oligonucleotides were synthesized from Invitrogen (Grand Island, NY, USA). The probe was 5′-end-labeled with [**γ**-32p]ATP (New England Nuclear Life Science Products, Boston, MA, USA), and the end-labeled probe was purified with a G50 column (Roche Molecular Biochemicals, Indianapolis, IN, USA). Approximately 2 × 10^5^ cpm of end-labeled DNA probe was incubated with 5 μg of nuclear extract on ice for 30 min. Protein–DNA complexes were electrophoresed on 4% polyacrylamide gel at 30 mA for 100 min in TBE running buffer (1.0 m Tris, 0.9 m boric acid, 0.01 m EDTA). The gel was transferred to Whatman paper, vacuum-dried, and scanned by a STORM PhosphorImager.

### Electrophoretic mobility shift assay coupled with Western blot

EMSA coupled with Western blot was performed as described previously (Quan *et al*., 2005). Briefly, the major shifted bands were excised from the EMSA gel and were then resolved in extraction buffer (1X PBS, 10 mm Tris-HCl, 1% Nonidet P-40, 1% sodium deoxycholate, 0.5% SDS, 2 μg/mL leupeptin, and 100 μg/mL phenylmethylsulfonyl fluoride) overnight at 37 °C. The gel debris was removed by centrifugation, and the supernatant was concentrated by Centricon-10 tubes (Amicon, Inc., Beverly, MA, USA). The samples were then heated for 5 min at 95 °C and subjected to SDS-PAGE and Western blot.

### Atomic force microscopy (AFM) imaging

The mechanical properties of cells were measured by AFM using our established techniques with minor modifications (Quan *et al*., 2012; Thomas *et al*., 2013). Briefly, monolayer cells or collagen gels were washed with PBS at least three times. The cell mechanical properties of traction force and elastic modules/tensile strength were measured by the Dimension Icon AFM system (Bruker-AXS, Santa Barbara, CA, USA) using PeakForce Quantitative NanoMechanics mode in fluid condition using a silicon AFM probe (PPP-BSI, force constant 0.01–0.5 N/m, resonant frequency 12–45 kHz, NANOSENSORS™, Neuchatel, Switzerland). PeakForceTM Quantitative Nanomechanical Mapping (QNMTM) is a new AFM technique for measuring nanoscale mechanical properties by calculation of Young's modulus of materials with high spatial resolution and surface sensitivity. In this mode, the probe is oscillated at a low frequency (1–2 kHz), capturing a force curve each time the AFM tip taps on the sample's surface. These force curves are then analyzed in real-time calculation of Young's modulus at each surface contact (Thomas *et al*., 2013). Nanoscale collagen fibrils were imaged by the AFM ScanAsyst mode in air using a cantilever (NSC15/AIBS; MikroMasch, San Jose, CA, USA) with a full tip cone angle ∼40 ° and a tip radius of curvature ∼10 nm. AFM was conducted at the Electron Microbeam Analysis Laboratory (EMAL) of the University of Michigan College of Engineering and was analyzed using Nanoscope Analysis software (Nanoscope Analysis v120R1sr3, Bruker-AXS, Santa Barbara, CA, USA).

### Statistical analysis

Statistical significance between groups was determined with the Student's *t*-test. All *P* values are two-tailed and considered significant when *P* < 0.05.
